# Voxel-Based Neighborhood for Spatial Shape Pattern Classification of Lidar Point Clouds with Supervised Learning

**DOI:** 10.3390/s17030594

**Published:** 2017-03-15

**Authors:** Victoria Plaza-Leiva, Jose Antonio Gomez-Ruiz, Anthony Mandow, Alfonso García-Cerezo

**Affiliations:** Grupo de Investigación de Ingeniería de Sistemas y Automática, Andalucía Tech, Universidad de Málaga, 29071 Málaga, Spain; victoriaplaza@uma.es (V.P.-L.); amandow@uma.es (A.M.); ajgarcia@uma.es (A.G.-C.)

**Keywords:** 3D laser scanner, spatial shape features, 3D classification, point clouds, voxels, supervised learning, neural networks, lidar, ground vehicles

## Abstract

Improving the effectiveness of spatial shape features classification from 3D lidar data is very relevant because it is largely used as a fundamental step towards higher level scene understanding challenges of autonomous vehicles and terrestrial robots. In this sense, computing neighborhood for points in dense scans becomes a costly process for both training and classification. This paper proposes a new general framework for implementing and comparing different supervised learning classifiers with a simple voxel-based neighborhood computation where points in each non-overlapping voxel in a regular grid are assigned to the same class by considering features within a support region defined by the voxel itself. The contribution provides offline training and online classification procedures as well as five alternative feature vector definitions based on principal component analysis for scatter, tubular and planar shapes. Moreover, the feasibility of this approach is evaluated by implementing a neural network (NN) method previously proposed by the authors as well as three other supervised learning classifiers found in scene processing methods: support vector machines (SVM), Gaussian processes (GP), and Gaussian mixture models (GMM). A comparative performance analysis is presented using real point clouds from both natural and urban environments and two different 3D rangefinders (a tilting Hokuyo UTM-30LX and a Riegl). Classification performance metrics and processing time measurements confirm the benefits of the NN classifier and the feasibility of voxel-based neighborhood.

## 1. Introduction

Three-dimensional (3D) lidar sensors are a key technology for navigation, localization, mapping and scene understanding in novel ground vehicle systems such as autonomous cars [1], search and rescue robots [2], and planetary exploration rovers [3]. One major limitation regarding the use of lidar technology in these challenging applications is the time and computational resources required to process the dense point clouds generated by these sensors.

Classification techniques involving point clouds are used extensively and can be categorized in many ways [4]. For instance, airborne sensors can use elevation and flatness characteristics to classify roof surfaces and urban objects [5,6,7], whereas terrestrial scans are affected by obstructions and varying point density [8]. Furthermore, algorithms have been proposed to identify particular object types, such as vehicles, buildings or trees [9,10], or to classify geometric primitives at point level [11]. In this sense, while some methods segment the cloud before classifying points within the resulting clusters [12,13], others perform classification directly on scan points [8]. Moreover, different machine learning descriptors have been considered (e.g., histograms [4,8] and conditional random fields [14,15]). In particular, many solutions rely on supervised learning classifiers such as Support Vector Machines (SVM) [12,16,17,18], Gaussian Processes (GP) [19,20], or Gaussian Mixture Models (GMM) [11,21,22,23].

This work focuses on improving the effectiveness, both in computational load and accuracy, of supervised learning classification of spatial shape features (i.e., tubular, planar or scatter shapes) obtained from covariance analysis [11]. This is very relevant because classification of primitive geometric features is largely used as a fundamental step towards higher level scene understanding problems [9]. For instance, classifying points into coarse geometric categories such as vertical or horizontal has been proposed as the first layer of a hierarchical methodology to process complex urban scenes [24]. Furthermore, classification of scan points prior to segmentation is useful to process objects with unclear boundaries, such as ground, vegetation and tree crowns [8]. In this sense, spatial shape features can describe the shape of objects for later contextual classification [15]. Thus, classification of spatial shape features based on principal component analysis (PCA) is a constituent process in recent scene processing methods [4,13,18,23,25].

Many classification techniques are point-wise in that they compute features for every point in a cloud by using the points within its local neighborhood, the support region. The *k*-Nearest Neighbors (KNN) algorithm can produce irregular support regions whose volume depends on the varying sampling density of objects and surfaces from terrestrial scans [8]. For example, KNN has been used to compare the performance between several classifiers [12] and to classify into planar or non-planar surfaces [18]. The KNN support volume can be limited by setting a fix-bound radius [26]. Furthermore, ellipsoidal support regions of adaptive sizes, denoted as super-voxels, can be built iteratively based on point characteristics [14,27]. Other point-wise classification techniques adopt regular support regions by searching for all neighbors within a given radius [8,9,11,20]. In general, point-wise techniques imply a high computational load. This is why some authors have proposed oversampling techniques to reduce the amount of data in the raw point cloud [14,28].

Grid representations and voxels have also been considered to speed up point cloud classification. In some solutions, grids serve to segment points prior to point-wise classification. For instance, the method proposed in [26] computes segmentation by projecting non ground points on a 2D grid and [29] uses voxels for defining groups of points that are later classified with a Neural Network (NN) supervised learning method. Some authors have proposed computing features for points in a voxel by considering support regions defined by neighboring voxels. The authors in [4] compute PCA for each voxel with a support region defined by the 26-neighbors. Descriptors are used both for segmentation (i.e., voxel clusters) and for later classification of the set of points within a cluster. Furthermore, in [13], the feature vector for each voxel is obtained from a support region that includes a number of surrounding voxels. In this case, features are not employed for classification but for mapping voxels to a color space used for segmentation. Neither [4] nor [13] compute features to classify points within a voxel.

In a previous work [30], we proposed an NN supervised learning formalism for classification of spatial shape features in lidar point clouds. Our interest was to use this classification method for object segmentation [31] and construction of 2D occupancy grids for autonomous navigation [32]. In order to reduce the computational load of the NN classifier in [30], we implemented a computationally simple voxel-based neighborhood approach where all points in each non-overlapping voxel in a regular grid were assigned to the same class by considering features within a support region defined only by the voxel itself. This work advanced promising classification results in a natural environment according to visual validation by a human expert. These preliminary results demand further analysis of the NN method with performance metrics and considering other types of environments and sensors. More importantly, it would be interesting to generalize voxel-based neighborhood so that it can be used with other supervised classifiers.

This paper extends [30] by addressing these questions. In particular, we analyze the NN classification method by proposing a new general framework for implementing and comparing different supervised learning classifiers that develops the voxel-based neighborhood concept. This original contribution defines offline training and online classification procedures as well as five alternative PCA-based feature vector definitions. We focus on spatial shape classes usually found in literature: scatter, tubular, and planar. In addition, we evaluate the feasibility of the voxel-based neighborhood concept for classification of terrestrial scene scans by implementing our NN method and three other classifiers commonly found in scene classification applications: SVM, GP, and GMM. A comparative performance analysis has been carried out with experimental datasets from both natural and urban environments and two different 3D rangefinders (a tilting Hokuyo and a Riegl). Classification performance metrics and processing time measurements confirm the benefits of the NN classifier and the feasibility of voxel-based neighborhood.

The rest of the paper is organized as follows. The next section reviews supervised learning methods that will be considered in the comparative analysis. Then, Section 3 proposes a general voxel-based neighborhood approach for supervised learning classification of spatial shape features. Section 4 describes the experimental setup and methodology for performance analysis offered in Section 5, which discusses results for different classifiers and feature vector definitions. The paper closes with the conclusions section.

## 2. Supervised Learning Methods for Point Cloud Classification

This section briefly reviews supervised learning methods that have been used in the literature for point cloud scene classification: SVM, GP, GMM, and NN.

### 2.1. Support Vector Machine

The purpose of SVM learning [33] is to find a hyperplane that separates the dataset into a discrete predefined set of classes consistent with labeled training patterns. When patterns are not linearly separable, SVM transforms original data into a new space and uses a kernel function for classification. SVM has shown good generalization even with a reduced training dataset, although its performance can be significantly affected by parametrization [34]. Apart from the definition of the kernel function, SVM uses a box constraint, which is a parameter that controls the maximum penalty imposed on margin-violating observations and contributes to prevent overfitting.

SVM has been applied to classify urban point clouds into ground, and planar and non-planar points on the ground [18]. In this application, every point is evaluated together with its KNN based on covariance analysis that uses a linear combination of eigenvalues and a Radial Basis kernel function. Furthermore, the same kernel function with SVM has been applied to lidar data in intelligent vehicles to detect vegetation [17] and to classify clusters of points as urban objects [4,12,16].

### 2.2. Gaussian Processes

GP is a generalization of the Gaussian probability distribution [35] that can be interpreted as a Bayesian version of the SVM method. Each class is modeled as a GP where a covariance function (kernel) is trained to estimate its nonparametric underlying distribution. The problem of learning in GP is exactly the problem of finding suitable parameters (called hyperparameters) for the covariance and mean functions that best model the training input dataset. Generally, the GP method requires defining the following: the number of function evaluations, a covariance function, an inference method, a mean function, a likelihood function, and the initialization of the hyperparameters.

GP has been applied for real-time ground segmentation by considering the relative height of lidar points from a land vehicle [19]. Moreover, a combination of GP and SVM with PCA has been proposed to classify terrain as traversable or non traversable by computing two features representing texture and slope for every point [20].

### 2.3. Gaussian Mixture Model

A GMM is a probabilistic model that uses a mixture of Gaussian probability distributions to represent subpopulations within a population [36]. In the case of more than two classes, a different GMM is inferred for each class. Then, the learning algorithm tunes the weight, mean, and covariance matrices of a mixture of $n_{g}$ Gaussian components for each GMM. The training process finds $n_{g}$ for each GMM given a maximum value $N_{G}$.

Lalonde et al. [11] used GMM with the expectation maximization (EM) algorithm [37] to classify lidar points in scatter, planar and tubular classes according to saliency features. GMM has also been applied with color and spatial features for pixel-wise segmentation of road images [21] and object and background classification in point clouds [22].

### 2.4. Neural Networks

The multi-layer perceptron (MLP) is a type of NN commonly used in supervised learning [38]. Implementing an MLP requires a definition of the network topology (i.e., the number of layers and neurons), the transfer function in every layer, the back-propagation learning algorithm, and the learning constant.

MLPs have been used to classify urban objects from non-ground points distributed within point clusters [25] and voxels [29]. Furthermore, we proposed an MLP formalism for classifying spatial shape features from natural environments [30]. Besides, the problem of classifying vehicles represented as point clouds has been addressed with a combination of NN and genetic algorithms [39].

## 3. General Voxel-Based Neighborhood Framework for Geometric Pattern Classification

This section proposes a voxel-based geometric pattern classification approach which can be generally used by supervised learning methods. General offline training and online classification procedures are detailed. Moreover, five alternative feature vector definitions are given to classify voxels as three spatial shape classes: scatter, tubular, and planar. Furthermore, data structures are proposed for the implementation of the point cloud and the input dataset.

### 3.1. Definitions

In general, classifiers produce a score to indicate the degree to which a pattern is a member of a class. For an input space with *N* patterns, the input dataset is defined as $D=\{\left(p_{i},t_{i}\right)|\forall i\in \left[1,N\right]\}$, where $p_{i}=\left[p_{i1},...,p_{iN_{L}}\right]$ is the *i*th input pattern and $t_{i}\in C$ represents one of the $N_{C}$ target classes, with $C=\{C_{1},...,C_{N_{C}}\}$. The $N_{L}$ components of $p_{i}$ are computed according to a feature vector definition F. Supervised learning needs a training dataset whose $p_{i}$ have been previously labeled with their corresponding $t_{i}$.

In this work, the goal is to classify scene points into three classes (i.e., $N_{C}=3$): $C=\{C_{1},C_{2},C_{3}\}$, where $C_{1}$, $C_{2}$, and $C_{3}$, correspond to scatter, tubular and planar shapes, respectively. By using voxel-based neighborhood, all points within a voxel are assigned to the same class. With this aim, the point cloud in Cartesian coordinates is voxelized into a 3D grid of regular cubic voxels of edge *E*. Edge size depends on the scale of the spatial shapes to be detected in the point cloud. Only those voxels containing more points than a threshold *ρ* are considered to be significant for classification. Thus, the size *N* of the input dataset is the number of significant voxels.

### 3.2. Training and Classification Procedures

**Figure 1:**
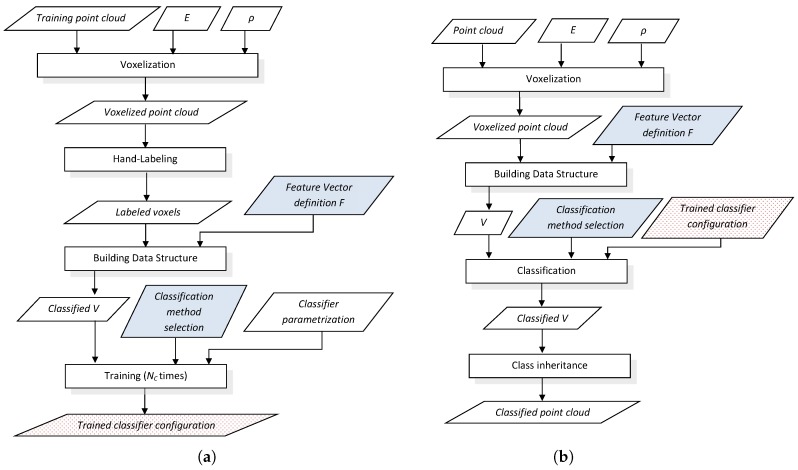
Offline training (**a**) and online classification (**b**) procedures with voxel-based neighborhood computation. The choices of a feature definition and a classification method are common for both procedures (shaded in solid blue). The trained classifier configuration (shaded in dotted red) output in (**a**) is used in (**b**).

General training and classification procedures particularized for voxel-based neighborhood are shown in Figure 1. Training is an offline process that has to be done once for a given classifier, whereas classification is performed online for each new point cloud. The training procedure produces a multi-class classifier configuration consisting on a set of $N_{C}$ classifiers that will be used in the classification procedure. Moreover, the choice of a feature vector definition and a particular classification method must be the same for the training and classification procedures.

A data structure *V* is defined to contain the input dataset $D=\{\left(p_{i},t_{i}\right)\}$. When all $t_{i}$ values in *V* have been set, either manually or automatically, this is considered a “classified *V*”. An implementation of *V* is described in Section 3.4.

The training procedure (see Figure 1a) uses a point cloud in Cartesian coordinates where the $N_{C}$ geometric classes must be represented and discernible. After voxelization, the *N* significant voxels in the 3D grid are manually labeled with their corresponding class ($t_{i}$) by a human supervisor. Then, a classified *V* data structure is built from the labeled voxels by computing $p_{i}$ for a particular choice of feature vector definition F (e.g., one of the definitions proposed in Section 3.3). Training is performed for a given classification method with its particular parameters, where a different configuration is inferred for each class. The output of the training procedure is the trained classifier configuration.

The goal of the online classification procedure (see Figure 1b) is to classify a new point cloud. The voxelized point cloud is used to create the *V* data structure with $p_{i}$ values computed with the same feature vector definition as in the training procedure. In the classification step, the trained classifier configuration given by the training procedure completes the classified *V* by appending $t_{i}$ values computed by considering the highest score of the $N_{C}$ classifiers. With voxel-based neighborhood, the classification for each voxel is inherited by all points within its limits.

### 3.3. Extracting Spatial Shape Features from Voxels

The local spatial distribution of all the points within a voxel is obtained as a decomposition in the principal components of the covariance matrix from point Cartesian coordinates. These principal components or eigenvalues are sorted in ascending order as $\lambda_{0}\geq \lambda_{1}\geq \lambda_{2}$ [11].

A feature vector F consisting on a linear combination of the eigenvalues [11] and $N_{L}=3$ is generally considered in the literature [13,17]:

(1) $$F=[\lambda_{0},\lambda_{0}-\lambda_{1},\lambda_{1}-\lambda_{2}].$$

This definition takes into account that scatterness has no dominant direction ($\lambda_{0}\approx \lambda_{1}\approx \lambda_{2}$), tubularness shows alignment in one dominant direction ($\lambda_{0}\gg \lambda_{1}\approx \lambda_{2}$), and planarness has two dominant directions ($\lambda_{0}\approx \lambda_{1}\gg \lambda_{2}$).

Nevertheless, classifier convergence and performance can be affected by the definition and scaling of F [40]. Thus, variants of Equation (1) based on the normalization and linear combination of eigenvalues could improve the performance of a particular classifier. Particularly, five feature vector definitions are considered in this work:

- $F_{1}=\left[\lambda_{0},\lambda_{1},\lambda_{2}\right]$: eigenvalues from the covariance matrix.
- $F_{2}=\left[\lambda_{0},\lambda_{0}-\lambda_{1},\lambda_{1}-\lambda_{2}\right]$: linear combination of the eigenvalues, as in Equation (1).
- $F_{3}=\left[\bar{\lambda_{0}},\bar{\lambda_{1}},\bar{\lambda_{2}}\right]$: normalized eigenvalues.
- $F_{4}=\left[\bar{\lambda_{0}},\bar{\lambda_{0}-\lambda_{1}},\bar{\lambda_{1}-\lambda_{2}}\right]$: normalization of the linear combination.
- $F_{5}=\left[\bar{\lambda_{0}},\bar{\lambda_{0}}-\bar{\lambda_{1}},\bar{\lambda_{1}}-\bar{\lambda_{2}}\right]$: linear combination of normalized eigenvalues.

In $F_{3}$, $F_{4}$, and $F_{5}$, the overline over a value *c* denotes normalization of this value in [0, 1] with respect to a 95% confidence interval. This normalization is computed as follows:

(2) $$\bar{c}=\frac{c-\min\{c_{k}\}}{\max\left\{c_{k}\right\}-\min\left\{c_{k}\right\}},\;with\;c\in \left\{c_{k}\;|k=1,...,N_{95c}\right\},$$

where $N_{95c}$ represents the rounded integer number of the 95% significant voxels in the middle of the distribution of *c*.

The input patterns $p_{i}$ in D are computed by using the selected F definition with the eigenvalues given by the covariance matrix corresponding to the points within the *i*th significant voxel.

### 3.4. Data Structures

In order to represent D, the classification data structure *V* must be related to a list of Cartesian point cloud coordinates *C*. Particularly, efficient access to the list of points within each voxel is required both to compute the input patterns $p_{i}$ and to inherit classification by scan points. With this purpose, this section proposes two data structures that implement the point cloud *C* and *V*, respectively.

Then, *C* is defined as a sorted list of all scan points, where the *j*th element has the following data:

- $\left(x_{j},y_{j},z_{j}\right)$, the Cartesian point coordinates.
- $I_{j}\in \left[1,N_{V}\right]$, a scalar index of the voxel that contains the point. Assuming that a spatial 3D grid with $N_{V}$ voxels includes all scan points, then a unique natural number $I\in [1,N_{V}]$ can be associated to each voxel [41]. This index is associated to the point in the voxelization process.
- $t_{j}\in \left[1,N_{C}\right]$, a natural number representing the target class. This value is hand labeled in the training process and is the resulting class in the classification process.

The structure *V* that implements D is defined as a list of *N* elements, where the *i*th element corresponds to a significant voxel and contains:

- $I_{i}\in \left[1,N_{V}\right]$, the scalar index associated to the voxel,
- $p_{i}$, feature vector values to be used as input pattern,
- $t_{i}\in \left[1,N_{C}\right]$, a natural number representing the target class.

The computation of these data structures is as follows. First, all scan points in *C* are indexed with their corresponding voxel index, which is also used to sort the list. After that, if there are more than *ρ* consecutive elements in *C* with the same index number, then a new entry for that voxel is created in *V*. After voxel classification, points in *C* with the same voxel index inherit the target class of the corresponding voxel in *V*. Points in non-significant voxels will remain unclassified (i.e., with a null value in the target class field).

## 4. Experimental Setup and Methodology

This section describes the training and evaluation datasets, the parametrization of classifiers, and the methodology used for the comparative performance analysis offered in Section 5.

### 4.1. Experimental Datasets

**Figure 2:**
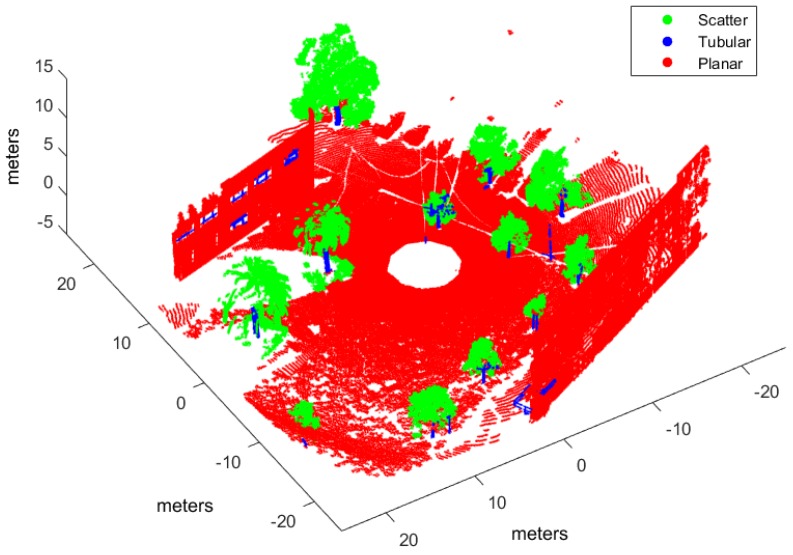
Hand labeled *Urban* point cloud.

**Figure 3:**
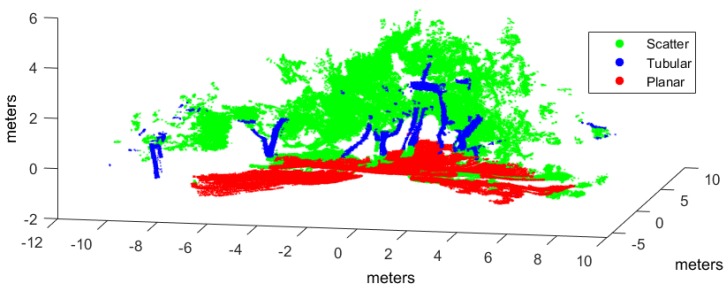
Hand labeled *Natural_1* point cloud.

**Figure 4:**
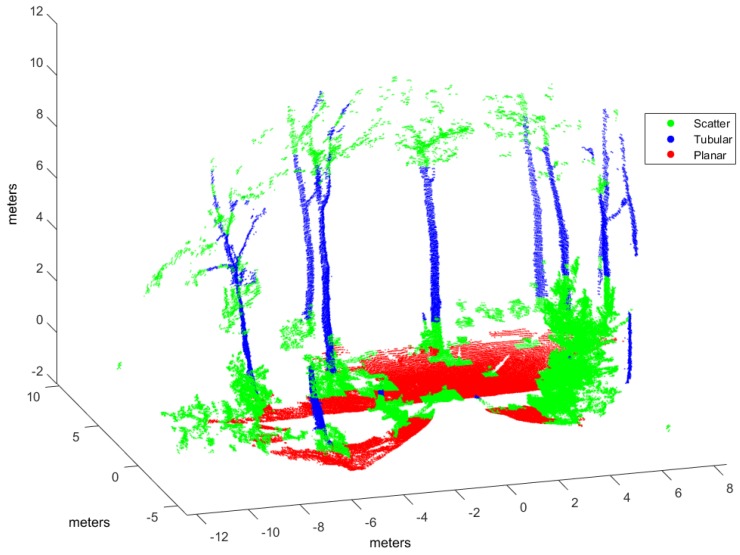
Hand labeled *Natural_2* point cloud.

Classification has been applied to three evaluation point clouds obtained with representative sensors and illustrative of natural and urban environments:

- *Urban*. This point cloud of a urban environment (see Figure 2) is a subset of the Sydney Campus dataset [42], which was scanned by a Riegl sensor (Horn, Austria). This is a complex scene which involves structured objects, mostly planes, such as buildings and flat floors.
- *Natural_1* and *Natural_2*. These point clouds are dominated by unstructured objects such as bushes, trees and rough terrain. Both scenes were scanned on natural areas close to Universidad de Málaga by a UNOLaser rangefinder (Málaga, Spain). This sensor is based on pitching a 2D Hokuyo UTM-30LX (Osaka, Japan) [43], with a maximum range of 30 m and horizontal and vertical fields of view of 270° and 131°, respectively. The first scan is from a complex scene with dense tree crowns (see Figure 3) and the second includes both bushes and tall trees with visible trunks (see Figure 4).

**Figure 5:**
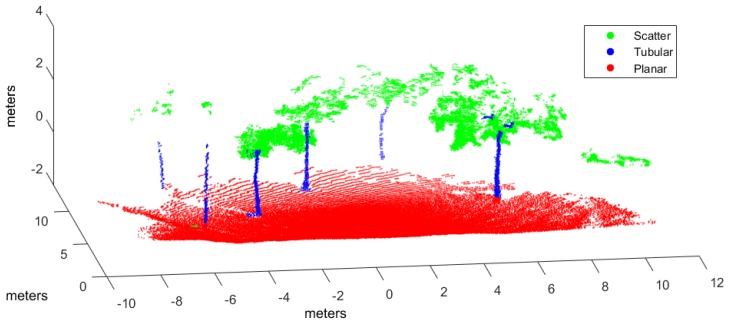
Hand labeled *Garden* point cloud.

As for the training procedure, a different point cloud has been considered:

- *Garden*. This point cloud contains elements from a semi-structured environment where the three geometric classes can be discernible for hand labeling: planar floor, tubular tree trunks, and scattered tree crowns (see Figure 5). This scene was scanned with the UNOLaser sensor.

Evaluation and training point clouds have been voxelized with E=0.5 m and ρ=10 (see Section 3.1), which were empirically determined [31]. Table 1 summarizes voxelization and hand labeling of experimental point clouds (evaluation datasets have also been hand labeled to evaluate classification performance). The table presents the resulting number of voxels and points included in the corresponding *V* structures, as well as the percentage of voxels for each class after hand labeling. In the *Urban* dataset, most voxels have been labeled as planar because clear floor and building walls dominate the scene. Conversely, in the *Natural_1* and *Natural_2* voxelized point clouds, a majority of the voxels are scatter or tubular due to bushes and trunks and treetops.

### 4.2. Classifiers Parametrization

The parametrization of the SVM classifier is the following:

- Function kernel: radial basis function as in [18],
- Box constraint: infinite.

The parameters used for the GP classifier are:

- Number of function evaluations: 30,
- Covariance function: squared exponential function with automatic relevance determination,
- Mean function: constant mean function,
- Inference method: expectation propagation algorithm,
- Likelihood function: cumulative Gaussian function,
- Hyperparameters of mean and covariance: 0 and (1,1,1,1), respectively (i.e., all length-scales and the signal magnitude are initialized to 1 and represented in the log space).

In GMM, the parameters are:

- $N_{G}^{scatter}=N_{G}^{tubular}=N_{G}^{planar}=10$,
- Marginal likelihood maximization: EM algorithm [37].

The proposed NN based classifier uses the following configuration:

- Network topology: multi-layer perceptron with one hidden layer of 100 neurons (this number was determined using a cascade learning constructive processing in which neurons are added to the hidden layer, one at time, until there is no further improvement in network performance),
- Transfer function: logistic transfer functions in hidden and output layers,
- Back-propagation learning algorithm: Levenberg–Marquardt,
- Learning constant: 0.02.

In addition, the training process of the NN must be stopped at an appropriate iteration to avoid overfitting. This iteration is found by the early stopping method of training [44], in which the training dataset is split into an estimation subset (80% of the training set) and a validation subset (the remaining 20%). More details of the configuration and implementation of the NN classifier can be found in [30].

### 4.3. Methodology

The performance of the classifiers will be compared by using classification statistical measures for each class. In particular, confusion matrices along with a multi-class extension of Matthew’s Correlation Coefficient (MCC) have been considered.

In a classification problem with $N_{C}$ target classes, a confusion matrix is the square matrix M ($N_{C}\times N_{C}$) whose ijth entry, $M_{ij}$, is the number of elements of true class *i* that have been assigned to class *j* by the classifier [45]. Therefore, an ideal classifier would yield a diagonal M. In this case, elements are points from significant voxels. Furthermore, in order to achieve a clear comparison between different datasets, normalized confusion matrices can be defined. Elements in the normalized confusion matrix $\bar{M}$ are defined as:

(3) $$\bar{M_{ij}}=\frac{M_{ij}}{\sum_{i=1}^{N_{C}}M_{ij}}\times 100,$$

where the sum of row elements is 100.

The generalization of MCC for the multi-class problem [46] is used as a reference performance measure on unbalanced datasets [45], which can be defined as follows:

(4) $$MCC=\frac{\sum_{i,j,k=1}^{N_{C}}M_{ii}M_{kj}-M_{ji}M_{ik}}{\sqrt{\sum_{i=1}^{N_{C}}\left[\left(\sum_{j=1}^{N_{C}}M_{ji}\right)\left(\sum_{m,n=1;m\neq i}^{N_{C}}M_{nm}\right)\right]}\sqrt{\sum_{i=1}^{N_{C}}\left[\left(\sum_{j=1}^{N_{C}}M_{ij}\right)\left(\sum_{m,n=1;m\neq i}^{N_{C}}M_{mn}\right)\right]}}.$$

MCC summarizes the confusion matrix into a single value in the [-1,1] range, where 1 represents a perfect classification and –1 extreme misclassification.

## 5. Performance Analysis and Comparison

This section discusses experimental results where the voxel-based approach proposed in Section 3 has been applied to the NN classifier and other supervised learning classifiers: SVM, GP, and GMM. First, all classifiers are compared with a representative feature vector definition. Then, an experimental analysis is performed to select an appropriate feature vector definition for each classifier. The section also includes a discussion of computation times as well as a comparison with a point-wise neighborhood classifier.

### 5.1. Performance Evaluation with Linear Combination of Eigenvalues

The evaluation datasets described in Section 4.1 have been used to compare the performance of the four classifiers trained with $F_{2}$, which is the feature vector definition given by Lalonde et al. [11]. Table 2 presents MCC and $\bar{M}$ for each classifier in all evaluations datasets. Regarding MCC, the NN classifier achieves the best results in all datasets. The GMM classifier obtains the second best performance, whereas SVM and GP get poor results. In particular, SVM never classifies patterns as class $C_{2}$ (tubular), as indicated by null values in the second column of $\bar{M}$ for all datasets. Similarly, GP classifies most points (over 90%) as class $C_{3}$ (planar). These results indicate poor performance of $F_{2}$ for some classifiers.

### 5.2. Performance Evaluation with Different Feature Vector Definitions

This section offers an experimental analysis to find a suitable selection of F for each classifier. With this purpose, all classifiers have been trained with the five feature vector definitions described in Section 3.3 using the *Garden* dataset. Table 3 summarizes this analysis by showing the corresponding MCC values. These results indicate that GP and SVM are strongly affected by the choice of the feature vector while GMM offers good results for all definitions. In this sense, the NN method achieves better results with the non-normalized definitions, which can be explained by the nonlinear qualities of the MLP. All in all, the best scores have been obtained with $F_{2}$ for NN, $F_{4}$ for GMM and GP, and $F_{5}$ for SVM. These definitions have been selected as the most appropriate choice for each classifier.

Comparative results with the corresponding F selections are given in Table 4. Regarding MCC, the NN classifier maintains the best results in all datasets. In addition, GP becomes the second best, clearly improving with respect to Table 2 (where it obtained the worst performance), which denotes the importance of an appropriate selection of F. As for $\bar{M}$, it can be noted that class $C_{2}$ (tubular) is the most difficult to classify (as indicated by low values of the $\bar{M_{22}}$ elements). In this difficult class, NN consistently outperforms all other classifiers and reaches 68.2% of true positives in the *Natural_2* dataset.

**Figure 6:**
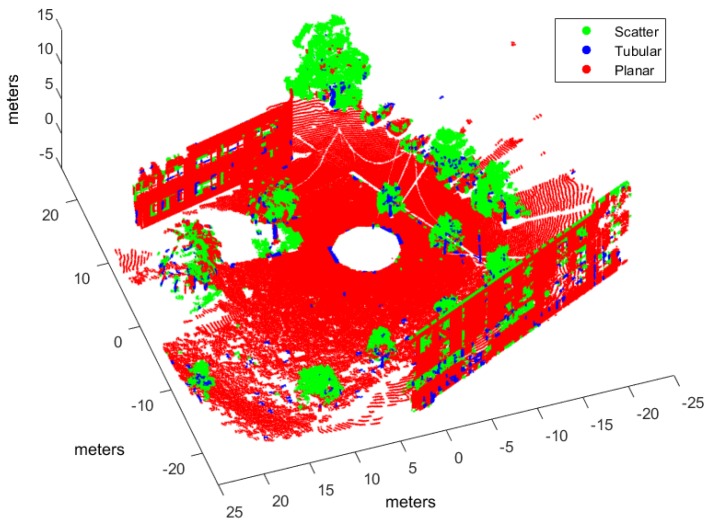
*Urban* point cloud classified by NN with $F_{2}$.

**Figure 7:**
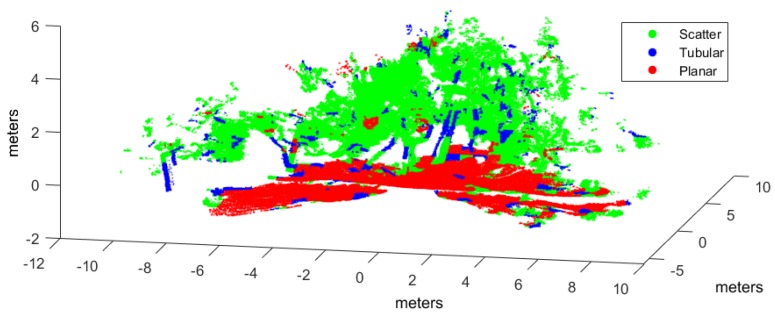
*Natural_1* point cloud classified by NN with $F_{2}$.

**Figure 8:**
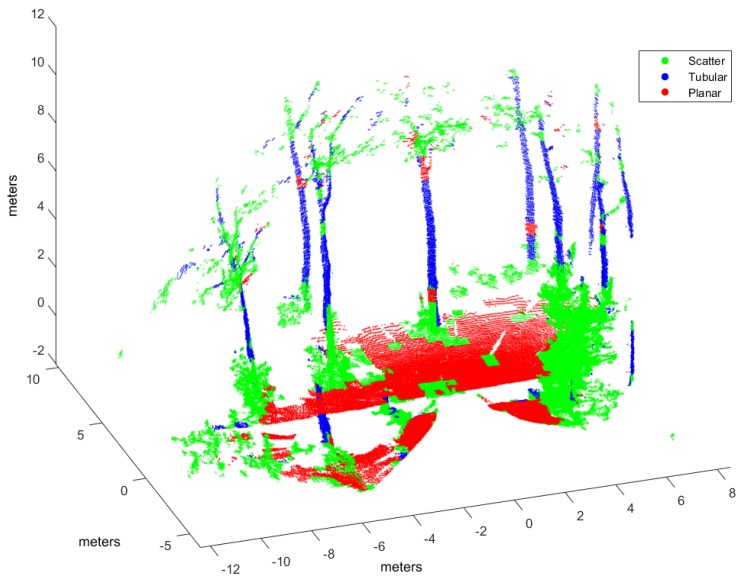
*Natural_2* point cloud classified by NN with $F_{2}$.

Figure 6, Figure 7 and Figure 8 illustrate the application of our NN classifier with the voxel-based neighborhood approach for the three evaluation datasets. These classification results show good accordance with the ground truth (i.e., hand labeled) values given in Figure 2, Figure 3 and Figure 4.

### 5.3. Computation Time

Table 5 presents execution times corresponding to a Matlab (R2015b, MathWorks, Natick, MA, USA) implementation of the classifiers running on a Core i7 processor with a clock frequency of 3.7 GHz and 16 GB of RAM. Computation of data structure *V* is common for all classifiers. Then, total computation time is obtained by adding the time for *V* computation to the training process time (in the offline procedure) or to the classification process time (in the online procedure).

*V* computation time includes voxelization as well as calculation of covariance matrices and their associated eigenvalues for every voxel. This value is proportional to the number of voxels in the data structure, which is greater for the *Urban* dataset (see Table 1).

Table 5 shows that GP requires much more computation time, for both training and classification, than the rest of classifiers. For offline training, the times for the training process, which offer considerable differences between the four classifiers, are greater than the time required for *V* computation. As for online classification, GMM, NN and SVM achieve classification times that are significantly faster than *V* computation, so their total computation times are similar and close to that value. Since the best classification performance in Table 4 was achieved by NN, it can be concluded that NN accomplishes an outstanding compromise between performance and computation time.

### 5.4. Comparison with Point-Wise Neighborhood Classification

Performance of voxel-based neighborhood has also been compared against point-wise neighborhood. In particular, the experimental datasets have been processed with a point-wise GMM classifier with $F_{2}$ (i.e., the configuration used by Lalonde et al. [11]) with a support region defined by a radius of 0.5 m. Classification performance and computation times are presented in Table 6 and Table 7, respectively.

Regarding classification performance, Table 6 presents MCC and $\bar{M}$ for point-wise GMM in all evaluation datasets. Comparing MCC values of Table 6 against the first row of Table 4, it can be appreciated that performance results are very similar. Particularly, voxel-based neighborhood outscores the point-wise method in the *Natural_2* and *Urban* datasets.

Total computation time is the sum of neighborhood computation and training/classification times, which are given as two separate rows in Table 7. In this case, most of the time is used for neighborhood computation. The comparison of this table with Table 5 shows that computation times for voxel-based neighborhood are dramatically reduced with respect to point-wise neighborhood.

In general, these results indicate that voxel-based neighborhood classification achieves a dramatic improvement in computation time with respect to point-wise neighborhood, while no relevant differences in performance can be appreciated. Furthermore, voxel-based NN has accomplished better classification performance with the experimental datasets.

## 6. Conclusions

Many point cloud classification problems targeting real-time applications such as autonomous vehicles and terrestrial robots have received attention in recent years. Among these problems, improving the effectiveness of spatial shape features classification from 3D lidar data remains a relevant challenge because it is largely used as a fundamental step towards higher level scene understanding solutions. In particular, searching for neighboring points in dense scans introduces a computational overhead for both training and classification.

In this paper, we have extended our previous work [30], where we devised a computationally simple voxel-based neighborhood approach for preliminary experimentation with a new a neural network (NN) classification model. Promising results demanded deeper analysis of the NN method (using performance metrics and different environments and sensors) as well as generalizing voxel-based neighborhood that could be implemented and tested with other supervised classifiers.

The originality of this work is a new general framework for supervised learning classifiers to reduce the computational load based on a simple voxel-based neighborhood definition where points in each non-overlapping voxel of a regular grid are assigned to the same class by considering features within a support region defined by the voxel itself. The contribution comprises offline training and online classification procedures as well as five alternative feature vector definitions based on principal component analysis for scatter, tubular and planar shapes.

Moreover, the feasibility of this approach has been evaluated by implementing four types of supervised learning classifiers found in scene processing methods: our NN model, support vector machines (SVM), Gaussian processes (GP), and Gaussian mixture models (GMM). An experimental performance analysis has been carried out using real scans from both natural and urban environments and two different 3D rangefinders: a tilting Hokuyo and a Riegl. The major conclusion from this analysis is that voxel-based neighborhood classification greatly improves computation time with respect to point-wise neighborhood, while no relevant differences in scene classification accuracy have been appreciated. Results have also shown that the choice of suitable features can have a dramatic effect on the performance of classification approaches. All in all, classification performance metrics and processing time measurements have confirmed the benefits of the NN classifier and the feasibility of the voxel-based neighborhood approach for terrestrial lidar scenes.

One additional advantage of processing each non-overlapping cell by using points from only that same cell is that this favors parallelization [47]. Developing a parallel version of the proposed method to improve online classification time with multi-core computers will be addressed in future work. Furthermore, it will be also interesting to adapt the method for incremental update of classification results with consecutive scans.

## Figures and Tables

**Table 1:** Characteristics of hand labeled voxels of experimental point clouds.

Dataset Type	Dataset	#Voxels	#Points	Voxels Percentage		
				Scatter	Tubular	Planar
Evaluation	*Urban*	13713	1473757	27.7	16.3	55.9
	*Natural_1*	1877	618913	72.8	6.2	20.9
	*Natural_2*	1346	267514	58.6	61.6	17.8
Training	*Garden*	974	128836	34.9	4.2	60.9

**Table 2:** Performance of classifiers (MCC and $\bar{M}$) using feature vector definition $F_{2}$ for the evaluation datasets.

	*Natural_1*		*Natural_2*		*Urban*	
	MCC	$\bar{M}$	MCC	$\bar{M}$	MCC	$\bar{M}$
GMM ($F_{2}$)	0.4631	$\left[\begin{array}{ccc} 74.3 & 18.7 & 7.0\\ 55.6 & 39.3 & 5.1\\ 14.2 & 9.2 & 76.6 \end{array}\right]$	0.5696	$\left[\begin{array}{ccc} 72.8 & 18.1 & 9.1\\ 18.3 & 65.4 & 16.3\\ 15.8 & 6.6 & 77.6 \end{array}\right]$	0.4962	$\left[\begin{array}{ccc} 81.5 & 11.7 & 6.8\\ 31.1 & 43.4 & 25.5\\ 11.9 & 15.0 & 73.1 \end{array}\right]$
GP ($F_{2}$)	0.0958	$\left[\begin{array}{ccc} 0.0 & 3.7 & 96.3\\ 0.0 & 6.1 & 93.9\\ 0.0 & 0.2 & 99.8 \end{array}\right]$	0.0005	$\left[\begin{array}{ccc} 0.0 & 1.3 & 98.7\\ 0.0 & 0.0 & 100\\ 0.0 & 0.0 & 100 \end{array}\right]$	0.0486	$\left[\begin{array}{ccc} 0.0 & 4.2 & 95.8\\ 0.0 & 2.9 & 97.1\\ 0.0 & 0.3 & 99.7 \end{array}\right]$
NN($F_{2}$)	0.6461	$\left[\begin{array}{ccc} 91.7 & 1.8 & 6.5\\ 56.4 & 37.6 & 6.0\\ 4.9 & 2.1 & 93.0 \end{array}\right]$	0.7927	$\left[\begin{array}{ccc} 95.9 & 2.3 & 1.8\\ 28.7 & 68.2 & 3.1\\ 6.9 & 1.0 & 92.1 \end{array}\right]$	0.6557	$\left[\begin{array}{ccc} 95.9 & 0.2 & 4.0\\ 11.7 & 22.9 & 65.4\\ 0.0 & 0.0 & 100 \end{array}\right]$
SVM($F_{2}$)	0.3091	$\left[\begin{array}{ccc} 48.6 & 0.0 & 51.4\\ 32.7 & 0.0 & 67.3\\ 0.8 & 0.0 & 99.2 \end{array}\right]$	0.1164	$\left[\begin{array}{ccc} 9.4 & 0.0 & 90.6\\ 2.2 & 0.0 & 97.8\\ 1.3 & 0.0 & 98.7 \end{array}\right]$	0.3152	$\left[\begin{array}{ccc} 43.2 & 0.0 & 56.8\\ 14.7 & 0.0 & 85.3\\ 0.1 & 0.0 & 99.9 \end{array}\right]$

**Table 3:** Performance of classifiers (MCC) for the training dataset with different definitions of the feature vector F.

	$F_{1}$	$F_{2}$	$F_{3}$	$F_{4}$	$F_{5}$
GMM	0.8667	0.8725	0.7856	0.9562	0.7988
GP	0.3414	0.0042	0.5996	0.8224	0.7420
NN	0.8295	0.8384	0.5659	0.4240	0.5633
SVM	0.0723	0.1687	0.4722	0.3275	0.5268

**Table 4:** Performance of classifiers (MCC and $\bar{M}$) for the evaluation datasets using selected feature vector definition ($F_{4}$ for GMM and GP, $F_{2}$ for NN, and $F_{5}$ for SVM).

	*Natural_1*		*Natural_2*		*Urban*	
	MCC	$\bar{M}$	MCC	$\bar{M}$	MCC	$\bar{M}$
GMM ($F_{4}$)	0.5352	$\left[\begin{array}{ccc} 94.4 & 2.8 & 2.8\\ 81.2 & 12.7 & 6.1\\ 13.1 & 1.1 & 85.8 \end{array}\right]$	0.5756	$\left[\begin{array}{ccc} 93.9 & 4.1 & 2.0\\ 66.7 & 27.4 & 5.9\\ 8.8 & 2.8 & 88.4 \end{array}\right]$	0.6021	$\left[\begin{array}{ccc} 97.4 & 1.3 & 1.3\\ 32.6 & 14.1 & 53.3\\ 2.6 & 0 & 97.4 \end{array}\right]$
GP ($F_{4}$)	0.5382	$\left[\begin{array}{ccc} 93.9 & 1.2 & 4.9\\ 64.2 & 9.6 & 26.2\\ 6.9 & 1.0 & 92.1 \end{array}\right]$	0.5871	$\left[\begin{array}{ccc} 96.3 & 0.8 & 2.9\\ 32.0 & 23.0 & 45.0\\ 7.4 & 2.7 & 89.9 \end{array}\right]$	0.6384	$\left[\begin{array}{ccc} 98.8 & 0.1 & 1.1\\ 13.6 & 15.3 & 71.1\\ 0.5 & 0 & 99.5 \end{array}\right]$
NN($F_{2}$)	0.6461	$\left[\begin{array}{ccc} 91.7 & 1.8 & 6.5\\ 56.4 & 37.6 & 6.0\\ 4.9 & 2.1 & 93.0 \end{array}\right]$	0.7927	$\left[\begin{array}{ccc} 95.9 & 2.3 & 1.8\\ 28.7 & 68.2 & 3.1\\ 6.9 & 1.0 & 92.1 \end{array}\right]$	0.6557	$\left[\begin{array}{ccc} 95.9 & 0.2 & 4.0\\ 11.7 & 22.9 & 65.4\\ 0.0 & 0.0 & 100 \end{array}\right]$
SVM ($F_{5}$)	0.4483	$\left[\begin{array}{ccc} 83.6 & 0.6 & 15.8\\ 54.7 & 0.8 & 44.5\\ 6.4 & 0.1 & 93.5 \end{array}\right]$	0.4646	$\left[\begin{array}{ccc} 86.9 & 1.3 & 11.8\\ 39.6 & 0.3 & 60.1\\ 6.8 & 0.0 & 93.2 \end{array}\right]$	0.5082	$\left[\begin{array}{ccc} 84.3 & 1.2 & 14.5\\ 20.7 & 0.7 & 78.6\\ 0.3 & 0.0 & 99.7 \end{array}\right]$

**Table 5:** Computation times for training and classification, in seconds.

	Training		Classification	
	*Garden*	*Urban*	*Natural_1*	*Natural_2*
*V* Computation	0.154	1.895	0.432	0.263
GMM	0.557	0.018	0.004	0.003
GP	104.673	3.995	4.010	3.795
NN	5.790	0.121	0.035	0.032
SVM	43.652	0.112	0.017	0.018

**Table 6:** Performance of GMM classifier with point-wise neighborhood using feature vector definition $F_{2}$ for the evaluation datasets.

	*Natural_1*		*Natural_2*		*Urban*	
	MCC	$\bar{M}$	MCC	$\bar{M}$	MCC	$\bar{M}$
$\begin{array}{c} \mathrm{Point}-\mathrm{wise}\\ \mathrm{GMM} \end{array}$	0.5392	$\left[\begin{array}{ccc} 79.2 & 7.6 & 13.2\\ 47.0 & 32.8 & 20.2\\ 6.9 & 1.9 & 91.2 \end{array}\right]$	0.5288	$\left[\begin{array}{ccc} 80.1 & 7.1 & 12.8\\ 16.7 & 33.3 & 50.0\\ 8.5 & 4.1 & 87.4 \end{array}\right]$	0.5797	$\left[\begin{array}{ccc} 79.4 & 6.6 & 14.0\\ 23.2 & 35.3 & 41.5\\ 3.3 & 1.2 & 95.5 \end{array}\right]$

**Table 7:** Computation times for point-wise neighborhood training and classification, in seconds.

	Training		Classification	
	*Garden*	*Urban*	*Natural_1*	*Natural_2*
*Neighborhood computation*	63.12	933.73	574.98	166.28
Point-wise GMM	8.89	9.29	1.36	0.63
